# Platelet Lysate Nebulization Protocol for the Treatment of COVID-19 and Its Sequels: Proof of Concept and Scientific Rationale

**DOI:** 10.3390/ijms22041856

**Published:** 2021-02-12

**Authors:** Maider Beitia, Diego Delgado, Pello Sánchez, Ana Vallejo de la Cueva, José Ramón Cugat, Mikel Sánchez

**Affiliations:** 1 Advanced Biological Therapy Unit, Hospital Vithas Vitoria, 01008 Vitoria-Gasteiz, Spain; maider.beitia@ucatrauma.com (M.B.); diego.delgado@ucatrauma.com (D.D.); pello.sanchez@ucatrauma.com (P.S.); 2 Care and Technology for Critical and Postcritical Patients Research Group, Bioaraba, 01009 Vitoria-Gasteiz, Spain; ANA.VALLEJODELACUEVA@osakidetza.eus; 3 Intensive Care Unit, Araba University Hospital, Osakidetza Basque Health Service, 01009 Vitoria-Gasteiz, Spain; 4 Pneumology Service, Sant Joan de Déu de Manresa Hospital, 08243 Manresa, Spain; pepepneumo@gmail.com

**Keywords:** platelet-rich plasma, platelet lysate, COVID-19, nebulization, platelet, growth factors

## Abstract

One of the most severe effects of coronavirus disease 2019 (COVID-19) is lung disorders such as acute respiratory distress syndrome. In the absence of effective treatments, it is necessary to search for new therapies and therapeutic targets. Platelets play a fundamental role in respiratory disorders resulting from viral infections, being the first line of defense against viruses and essential in maintaining lung function. The direct application of platelet lysate (PL) obtained from the platelet-rich plasma of healthy donors could help in the improvement of the patient due its anti-inflammatory, immunomodulatory, antifibrotic, and repairing effects. This work evaluates PL nebulization by analyzing its levels of growth factors and its biological activity on lung fibroblast cell cultures, besides describing a scientific basis for its use in this kind of pathology. The data of the work suggest that the molecular levels and biological activity of the PL are maintained after nebulization. Airway administration would allow acting directly on the lung tissue modulating inflammation and stimulating reparative processes on key structures such as the alveolocapillary barrier, improving the disease and sequels. The protocol developed in this work is a first step for the study of nebulized PL both in animal experimentation and in clinical trials.

## 1. Introduction

A new coronavirus named severe acute respiratory syndrome coronavirus 2 (SARS-CoV-2) was identified in China at the end of 2019 [1]. It is transmitted by saliva droplets and aerosols emitted by infected individuals [2]. People infected by this virus can progress either asymptomatically or develop the coronavirus disease 2019 (COVID-19) that can cause acute respiratory distress syndrome (ARDS) and/or cardiovascular complications among others which, in the most severe cases, might eventually lead to death [3,4,5,6,7]. Additionally, COVID-19 frequently produces sequels such as pulmonary fibrosis, which progressively reduces the respiratory capacity of patients and, hence, profoundly limits their quality of life [8,9]. Lastly, it is important to take into consideration the complications that can arise when generating immunity, since as in the previous SARS-associated coronavirus, antibodies against the patient’s own cells could be generated, developing immunopathological disorders [10].

While waiting for the effect of the new vaccines, there is no effective treatment to deal with this health emergency, and therefore, there is an urgent need for new treatments. The design of new therapies must be focused not only on the elimination of the virus but also on the recovery of the affected lung tissue and the avoidance or reduction of the severe sequels. In that sense, treatments based on regenerative medicine and specifically platelet-rich plasma (PRP) could be suitable and effective to tackle this disease.

Platelet-rich plasma, a biological therapy approved by regulatory agencies, is based on the concentration of the platelets present in blood [11]. Platelets are small anucleate cells derived from megakaryocytes whose biosynthesis takes place in both bone marrow and lungs [12,13]. Their actions rely not only on the regulation of thrombosis but also on the biological activity of the complex pool of chemokines, cytokines, and growth factors released from the platelet granules, which are able to induce biological processes of proliferative, antifibrotic, and anti-inflammatory nature. Thus, they have been classically considered the principal responsible of hemostasis, wound healing, anti-inflammatory activities, and tissue repair, among others [14]. However, recent research works have also shown an association of platelets with antiviral activities as well as lung biology and physiology [15]. Even if platelets are of great importance to activate antiviral defense, it has been revealed that viruses can also negatively affect platelets by translating viral RNA into proteins, which leads to a conversion of healthy platelets into detrimental ones [16].

Considering the mentioned pathophysiology generated by COVID-19 and other respiratory infections, PRP-based therapies could counteract the loss of healthy platelets by a local administration of healthy donors’ platelet content and consequently, retrieve and amplify their biological effects, boosting the regeneration of damaged lung tissue and the reduction of fibrosis. As that PRP must reach the alveoli in the lungs, we propose its inhaled application into the respiratory system by the aspiration of allogenic nebulized PRP. Therefore, the aim of this work is to develop a protocol to nebulize PRP and to evaluate its composition and bioactivity in order to apply it in COVID-19 patients to boost the recovery of the injured area as well as to prevent lung fibrosis sequels. In addition, a scientific basis for its use in this kind of pathologies is described.

## 2. Results

### 2.1. Platelet-Rich Plasma Characterization

**Figure 1:**
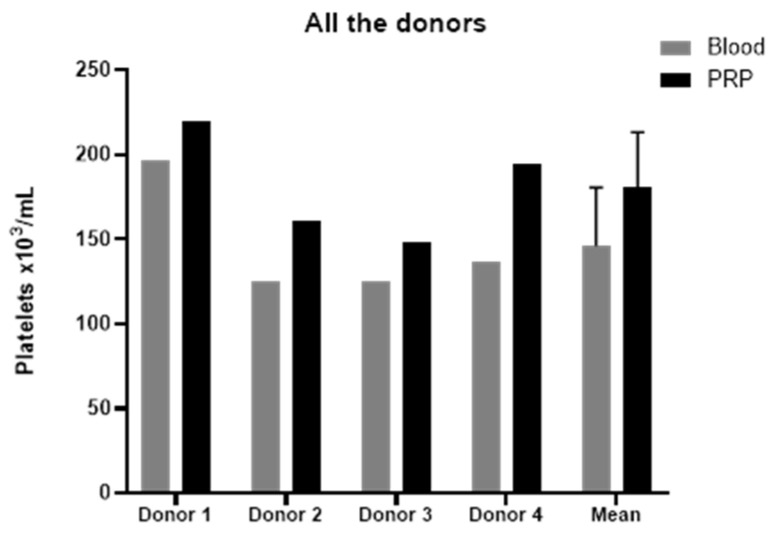
Platelet concentrations. Platelet concentration of blood and PRP of four donors and its mean value. Error bars = standard deviation. PRP: platelet-rich plasma.

The platelet-rich plasma (PRP) employed to elaborate platelet lysates presented a concentration of platelets slightly higher than blood levels (Figure 1), reaching a concentration factor of 1.24 ± 0.11, and with no leukocytes or erythrocytes. According to the latest coding system and minimum reporting requirements for PRP studies, the PRP used in this study was 11-00-11, and the characteristics of both PRPs are reported in Table 1 [17].

### 2.2. Growth Factor Profile in Standard and Nebulized Platelet-Rich Plasma 

**Figure 2:**
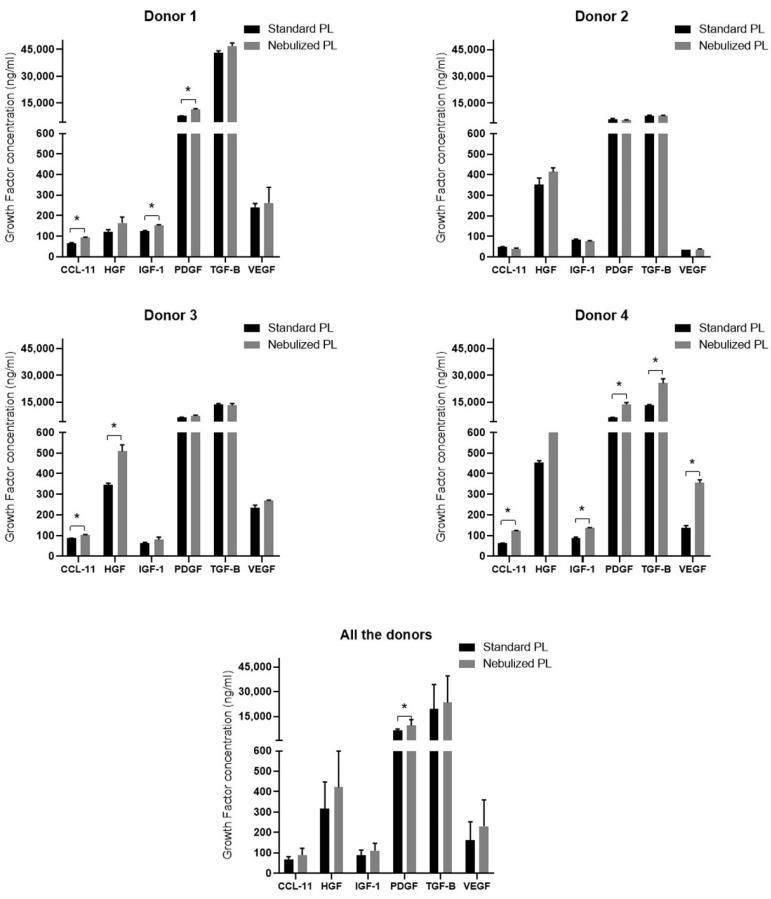
Soluble growth factor concentrations in platelet lysates. C-C Motif Chemokine Ligand 11 (CCL-11)/Eotaxin, Hepatocyte Growth Factor (HGF), Insulin-Like Growth Factor 1 (IGF-1), Platelet-Derived Growth Factor (PDGF), Transforming Growth Factor Beta (TGF-B), and Vascular Endothelial Growth Factor (VEGF) protein levels were measured in duplicate by enzyme-linked immunoabsorbent assay (ELISA) in the platelet lysates of four donors. Statistical significance of the differences between standard and nebulized platelet lysates were determined by *t*-test (* *p* < 0.05). PL: platelet lysate.

In order to assess whether standard and nebulized platelet lysates are similar in growth factors composition, enzyme-linked immunoabsorbent assays (ELISAs) were performed in the platelet lysates (PLs) of the four donors. The measured growth factors/cytokines and their reference range were as follows once the dilution factor is applied: C-C Motif Chemokine Ligand 11 (CCL-11) (15.6–1000 pg/mL), Hepatocyte Growth Factor (HGF) (156–10,000 pg/mL), Insulin-Like Growth Factor 1 (IGF-1) (94–600 ng/mL), Platelet-Derived Growth Factor (PDGF) (780–50,000 pg/mL), Transforming Growth Factor Beta (TGF-β) (1284–80,000 pg/mL), and Vascular Endothelial Growth Factor (VEGF) (31.3–2000 pg/mL). Results showed similar growth factor levels in both standard and nebulized PLs with a few statistically significant differences in some of the protein levels (Figure 2). All the differences between the two formulations followed the same tendency in the four donors, being the protein levels higher in the nebulized platelet lysate than in the standard one. However, donor 2 did not show any statistically significant differences in growth factor content.

When all the four donors were grouped in a single one, only PDGF showed statistically significant higher levels in the nebulized PL than in the standard one (Figure 2).

### 2.3. Impact of Platelet-Rich Plasma Nebulization on Its Bioactivity

**Figure 3:**
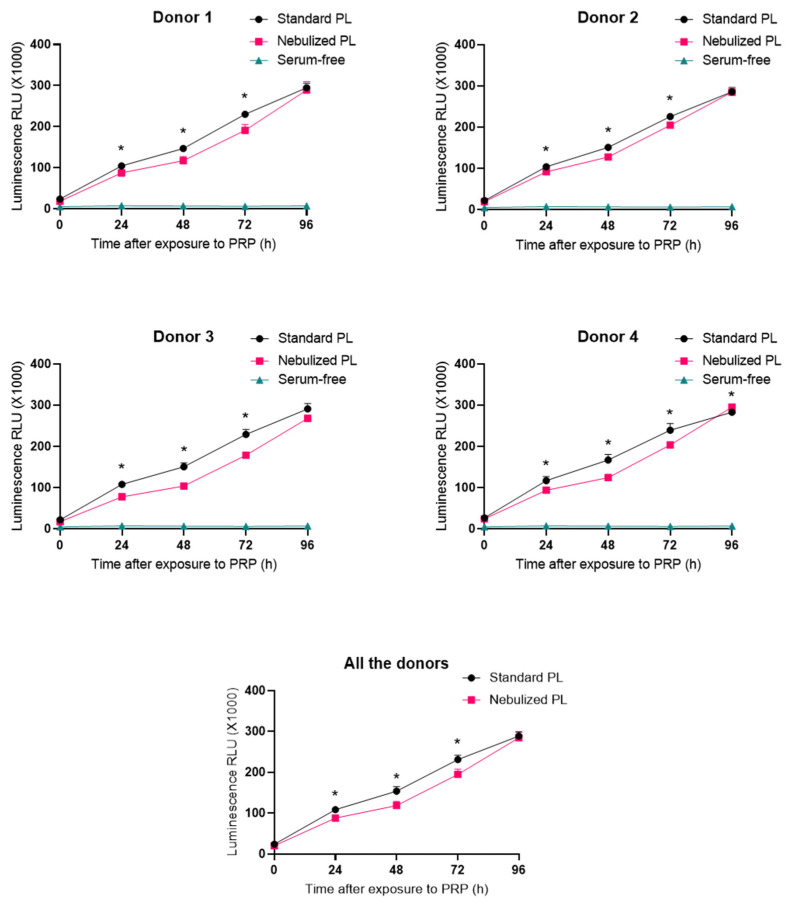
Growth curves of lung fibroblasts. Normal human lung fibroblasts were cultured with medium supplemented with either standard or nebulized platelet lysate in triplicate in four independent experiments, and cell viability was measured every 24 h. The statistical significance of the differences in proliferative capacity between standard and nebulized platelet lysates were determined by *t*-test (* *p* < 0.05). PL: platelet lysate.

With the aim of evaluating the bioactivity of standard and nebulized platelet lysates, real-time viability measurements were performed. Serum-free medium was used as the negative control. Both standard and nebulized platelet lysates triggered cellular growth over lung fibroblast throughout the days, while cells cultured in serum-free medium did not show any increase in cell viability (Figure 3). The differences between the standard and the nebulized PLs were significant usually at 48, 72, and 96 h timepoints. However, both formulations showed the same cellular growth tendency that was meaningfully superior to that observed in the serum-free condition.

## 3. Discussion

### 3.1. Role of Platelets against Viral Infections and SARS-CoV-2

Since 2019, the COVID-19 pandemic has challenged the health care system with an overwhelming number of cases, the most severe being vascular and respiratory that can lead to ARDS, profoundly threatening the life of the patient [18]. While the development of vaccines may help alleviate the pandemic situation, the search for new treatments and therapeutic targets is necessary both for infected patients and for those who have overcome the disease but present sequelae as well as for any infections affecting the respiratory system. In this work, we proved for the first time that the nebulization of platelet lysate does not affect its molecular levels and bioactivity on cell cultures, when cellular viability was measured. This opens the door to a potential use of PRP in a nebulized form and administrating the platelet content directly in the lungs.

Platelets are discoid and anucleated blood elements present at a concentration of 150,000–400,000 platelets/µL in the blood with a life span of 7 to 10 days. With a diameter of 2–3 µm, platelets have always been considered the main actors in the maintenance of hemostasis, wound healing, pathological development of thrombosis, and recently in the tissue repair processes. These processes are initiated by the activation of different platelet membrane receptors such as glycoproteins Ib (GPiB) and VI (GPVI). However, the platelet external plasma membrane presents a great number of receptors that trigger a network of intracellular signals that allow them to perform many other functions [19]. One of the most overlooked processes is its role in infections, and more specifically in viral infections, as was firstly shown by Terada et al. [20], who demonstrated the interaction of the influenza virus with platelets, working as carriers of the virus in the bloodstream. Since then, the number of scientific works has increased, confirming the key role of platelets in the first response against viral infections and their connection with the immune system [21].

The activation of platelet response is triggered by the interaction of the virus with the platelet, which is mediated by structures present in platelets and megakaryocytes called Pattern Recognition Receptors (PRRs). These receptors recognize molecular signals derived from both external pathogens (pathogen-associated molecular patterns, PAMPs) and products of tissue destruction (damage-associated molecular patterns, DAMPs). A large number of PRR types are grouped into recipient families such as Toll-like receptors (TLR), C-type lectin receptors (CLR), Dendritic Cell-Specific ICAM3-Grabbing Non-Integrin (DC-SIGN), complement receptors, or chemokine receptors [22]. It should be noted that although platelets are not nucleated cells, megakaryocytes provide them during their formation with both the genetic and molecular material needed to synthesize proteins, which can also be translated from RNA viruses [23]. In the case of the binding between SARS-CoV-2 and platelets, the results to date are contradictory. Manne et al. did not detect Angiotensin-Converting Enzyme 2 (ACE-2) receptors mRNA or protein neither in platelets nor in megakaryocytes of COVID-19 patients, suggesting that the virus uptake in platelets happens by alternative mechanisms [24]. However, other studies did demonstrate the presence of ACE-2 receptors on platelets, as well as the cellular serine protease TMPRSS2 that primes SARS-CoV-2 for cell entry [25,26]. In addition, Zhang et al. observed the internalization and degradation of the ACE-2 receptor after interaction with the virus. This could explain these contradictory results, since Manne et al. analyzed platelets coming mainly from COVID-19 patients, so the degradation of ACE-2 receptors would have already occurred.

**Figure 4:**
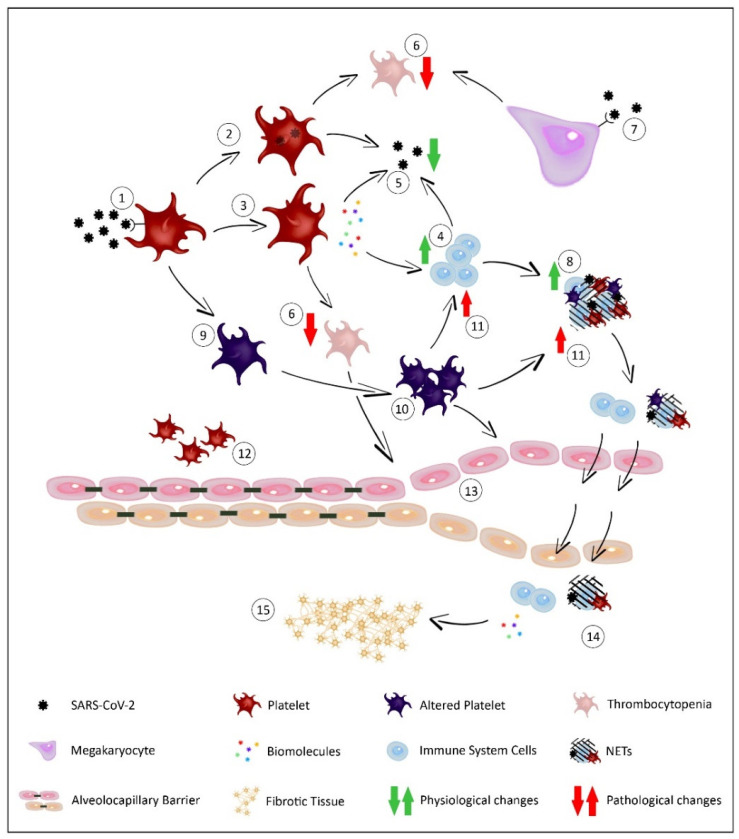
Platelet action in respiratory infections. After receptor-mediated binding between the platelet and the virus (**1**), different responses are triggered. Viral pathogens can be phagocytized by platelets (**2**), which are also activated producing a degranulation and release of different biomolecules (**3**). While some biomolecules have microbiocidal properties, others activate the cells of the immune system (**4**). All these actions cause both the elimination of the virus (**5**) and the destruction of platelets, causing a thrombocytopenia (**6**). This thrombocytopenia is also influenced by the decrease in the production of platelets by the megakaryocytes after their interaction with the virus (**7**). In addition, the activation of immune system cells such as neutrophils generates structures and aggregates such as Neutrophil Extracellular Traps (NETs) that help eliminate pathogens (**8**). However, the interaction of viruses with platelets alters both their structure and functionality (**9**), becoming more hyperreactive (**10**). As a consequence, the response of the immune cells is exaggerated and dysregulated (**11**), aggravating processes such as inflammation. The stabilizing function of the alveolocapillary barrier carried out by platelets (**12**) is also altered due to thrombocytopenia and platelet alteration (**13**). The destructuration of this barrier allows the passage of fluid, cellular aggregates and inflammatory molecules (**14**) into the lung tissue, generating edema and the acute respiratory distress syndrome (ARDS) symptomatology. Attempts at tissue repair during these processes are also disrupted, generating an accumulation of cellular polarities and extracellular matrix components such as collagen, leading to fibrosis (**15**). The application of the content from healthy donors directly in the lungs could modulate processes such as inflammation and repair, improving both the symptoms and the sequels of the patient.

Once the interaction of the platelet with the virus is achieved, different defense responses are produced, which can be direct or indirect through the immune system [27] (Figure 4). Concerning direct responses, platelets are able to phagocytize viral elements through endosomal and lysosomal mechanisms, resulting in the transport of pathogens and the clearance of the virus [28]. After binding the viral pathogens, the platelets can also be activated, leading to the degranulation and release of the contents stored in different granules called dense granules, α-granules, and lysosomes. Among the multitude of substances released, microbicide molecules such as peptides and cytokines are present, which confer antiviral activity to the platelets, helping inactivate and eliminate the virus [29]. This platelet degranulation and consequent release of molecules also generate an indirect response against infection, involving cells of the immune system [30,31,32,33].

After the interaction between virus and platelets and the responses generated, a series of consequences are unleashed, whose objective is the elimination of the threat. However, an imbalance in the response can lead to the worsening of the infection. Recent studies showed that this interaction and platelet subsequent activation to perform the defense against the infection cause genetic, structural, and functional alterations of the platelets, making them more hyperreactive and generating responses that can aggravate the patient’s condition. Middleton et al. found that viral infections altered the expression of numerous transcripts in human and murine platelets, upregulating the expression of the integrin alpha-IIβ (ITGA2B) that is associated with increased mortality [16]. The same group demonstrated for the first time that SARS-CoV-2 significantly altered both gene expression and functionality, leading to platelet hyperreactivity. Platelets of COVID-19 patients showed an increased expression of P-selectin as well as faster platelet aggregation together with an increased production of collagen and fibrinogen [24]. This may dysregulate and exacerbate the platelet-mediated immune response, worsening the pathology of these patients.

As mentioned above, this immune response is activated by the release of cytokines after the activation and subsequent degranulation of platelets. The presence of cytokines and chemokines such as CD40L, Interleukin 1-β (IL-1β), or platelet factor 4 (CXCL4) generate inflammation processes that affect endothelial cells, activation of the complement system, and recruitment of leukocytes in the attempt to eliminate pathogens [34,35,36,37]. Platelet alteration and hyperreactivity caused by virus interaction could lead to the dysregulated formation of Neutrophil Extracellular Traps (NETs) or NETosis. The interaction between platelets, pathogens, and neutrophils originate these structures consisting of DNA, decondensed chromatin, histones, and granule proteins. NETs are part of the innate immune system whose function is the trapping and elimination of pathogens, and they are involved in the inflammatory processes [15]. In infectious processes such as those caused by viruses, NETosis may be dysregulated and increased, leading to vascular and respiratory problems such as ARDS [38]. A recent study with COVID-19 patients found a strong correlation between the increased NETs formation and the augmented severity of the disease [39] and, along with other works, highlights the involvement of this dysregulated NETosis with ARDS and coagulopathy associated with COVID-19 [40,41]. In addition, more studies showed that SARS-CoV-2 induces the excessive formation of leukocyte-platelet aggregates, being present especially in COVID-19 patients who presented the most severe symptomatology [42]. These deregulated and inflammatory processes alter the alveolocapillary barrier, generating edemas that lead to pulmonary complications [25,42].

### 3.2. The Importance of Platelets in Lung Biology

Indeed, platelets play a fundamental role in the maintenance of the alveolocapillary barrier, which regulates the passage of fluids and cellular and molecular components from the systemic circulation into the alveolar space. This stabilization is produced thanks to factors released by the platelets such as sphingosine-1 that act on the endothelial cells, preserving this barrier [43]. In situations of inflammation and hyperreactivity of platelets generated in COVID-19, the disruption of this barrier occurs [15,44], allowing the passage of fluid and structures such as platelet aggregates and leukocyte–platelet aggregates to the alveolar space. As a result, edemas are produced, giving rise to the ARDS symptomatology [45]. Moreover, the destabilization of this barrier and the ultimate cause of ARDS also occurs in situations of thrombocytopenia that are present in this type of infection, creating a vicious circle [15]. Lippi et al. found a decrease in platelet count in COVID-19 patients that also resulted in a risk marker for increased mortality [46]. On the one hand, this thrombocytopenia is due to the elimination of the virus through platelet phagocytosis, which leads to platelet destruction. On the other hand, the degranulation of platelets in order to release their molecular content also contributes to thrombocytopenia. This reduction in platelet number is increased by the action of megakaryocytes that respond to the presence of viruses releasing interferon that causes a decline in platelet synthesis [42,47]. Finally, we must take into consideration the importance of the lung in the formation of platelets. While the classical idea stated that platelets were originated from megakaryocytes in the bone marrow and also into the bloodstream, this concept has evolved. Several works confirmed that megakaryocytes were capable of passing into the bloodstream and were trapped in the lungs, becoming important niches where these cells are accumulated and where they also carried out the release of platelets. This entrapment in the lungs is not only due to mechanical processes because of the size of megakaryocytes compared to the pulmonary blood vessels but also due to molecular processes of adhesion with the endothelium [48,49]. Thus, the affectation of the lungs would mean a decrease in the thrombopoiesis, which added to the processes of thrombocytopenia mentioned above, would aggravate the destabilization of the alveolocapillary barrier.

After the inflammation processes, destabilization of the alveolocapillary barrier and the consequent accumulation of the increased interstitial and intra-alveolar fluid and appearance of the ARDS, which compromises the gaseous exchange and the life of the patient, a reparative phase begins trying to repair the damaged tissue and restore the homeostasis. This phase is characterized by an expansion of resident fibroblasts and the formation of a provisional matrix as well as the proliferation of airway progenitor cells and type II alveolar epithelial cells, with differentiation into type I alveolar epithelial cells. Once epithelial integrity is reestablished, the resorption of alveolar edema and the temporary matrix restore the alveolar architecture and function. However, persistent damage due to inflammatory processes in the ARDS and failure of tissue repair lead to the development of interstitial changes and fibrosis. In this process, there is an accumulation of cell populations such as fibroblasts and macrophages as well as excess extracellular material, namely collagen and fibronectin. In addition, this excessive fibrosis is difficult to resolve due to the imbalance generated between antifibrotic and profibrotic factors [50]. This pathological process is one of the sequels that COVID-19 patients who have survived ARDS can suffer, meaning a decrease in the quality of life of patients as well as lung problems, fatigue, or shortened life expectancy [8].

### 3.3. Therapeutic Potential of Platelet-Rich Plasma against COVID-19

While developing a safe and effective vaccine and a vaccination plan that covers most of the population, it is important to have a therapeutic arsenal against the respiratory condition of COVID-19 as well as its sequels. Part of these treatments should be focused on the platelet response due to its importance in the defense against the virus and the lung biology, which are therapies not only effective against COVID-19 but also against other conditions that share this physiopathology and symptomatology. Among the treatments to be applied in these situations, those based on regenerative medicine should be taken into consideration due to their capacity to modulate inflammatory, immune, and reparative processes. Studies on mesenchymal stem cells (MSCs), which can come from different sources such as bone marrow, adipose tissue, or umbilical cord, showed their potential therapeutic effects on lung conditions caused by viral infections. Although many of its mechanisms remain unknown, researchers have highlighted the importance of the activation of MSCs through receptors such as TLRs, triggering a paracrine action through the release of several molecules: angiopoietin-1 and keratinocyte growth factor that favor the restoration of the alveolocapillary barrier in ARDS, chemokines such as the monocyte chemoattractant protein-1 (MCP-1) and the granulocyte-macrophage colony-stimulating factor (GM-CSF) that attract and activate leukocyte cells, exosomes, and anti-inflammatory molecules [51]. The first COVID-19 clinical trials with this therapy are offering promising results. The patients of these trials received human umbilical cord MSCs and bone marrow stem cell-derived exosomes by the intravenous route. Preliminary data suggest that the treatments are safe and well-tolerated, improving markers related to mortality, inflammation, and lung function [52,53,54].

Platelet-rich plasma is another treatment based on regenerative medicine that exploits the biomolecules present in both plasma and platelets to perform modulating actions on the altered biological processes. It is widely known that PRP has anti-inflammatory, immunomodulatory, angiogenic, anti-apoptotic, and anti-fibrotic properties [14,43,55,56,57,58]. Its stimulatory properties on different cell lines and the absence of general or local adverse effects on other tissues are also well known. In the last 20 years, its use has been extended to different pathologies of the locomotor system with very encouraging results in numerous pathologies, both acute and chronic. Situations that are difficult to treat, such as diabetic foot ulcers, have responded effectively to local treatment with PRP. PRP has already demonstrated its efficacy in the local treatment of degenerative conditions such as joint degeneration of the knee or hip [14]. It is also being applied in different pathologies in other branches of medicine such as ophthalmology, dermatology, nervous system lesions, and recently in gynecology as a treatment for ovarian and uterine pathology, for example. In all these pathologies, its efficacy and the ideal composition and dose of PRP are still being studied in multiple clinical studies [11].

In the case of patients affected by COVID-19 or other viral infections, the use of nebulized allogeneic PRP could be an alternative treatment. First, the administration of PRP by air would reach the lungs directly. The mode of application in other medicine fields is by local infiltration. In the case of lung pathology, we can only reach the broncho-alveolar tree by local instillation or nebulization. Instillation would provoke coughing in COVID-19 patients with a high risk of contagion by aerosolization. Therefore, the only way to deliver PRP into the alveoli would be nebulization. Second, autologous PRP could not be used due to the viral alteration of platelets, the significant increase in inflammatory cytokines present in plasma, and the thrombocytopenia associated with these pathologies. The application of the PRP effector molecules directly in the lung could favor an anti-inflammatory action through (1) molecules such as IGF-1 and HGF, which act on intracellular pathways [59], (2) the favoring of the reparative macrophages M2 phenotype [55], and (3) the action on oxidative stress [56]. Moreover, this modulation of the inflammation, which is key in the physiopathology of these infections, would be accompanied by an antiviral effect and by a tissue repair stimulation, allowing us to reestablish pulmonary structures as the alveolocapillary barrier. In fact, both in vitro and in vivo models have demonstrated the usefulness of PRP for the maintenance of the endothelial barrier, including the reversal of permeability in the lungs due to thrombocytopenia [60,61]. The different biomolecules present in platelet lysate modulate cellular activity, promoting tissue repair [57,62], which may also be favored by the reparative action of Alveolar Type II Cells, as they may mimic mesenchymal stem cells [63], and which, resembling these, could be stimulated by platelet molecules [64]. All these processes are based on the balanced action of the biomolecule cocktail present in the platelet lysate, which stimulate and inhibit different cellular response and interact with each other, complicating the cause–effect relationship (Table 2). The mechanism of action of biomolecules present in platelet lysate is based on their interaction with cell receptors, which triggers the different cellular signals that determine cell behavior. The resulting cellular response is both autocrine and paracrine, causing an effect not only on the cells stimulated by the platelet lysate but also on adjacent cells. This generates a global response throughout the tissue, making neither a direct intracellular action nor a complete diffusion of the platelet lysate in all tissue cells necessary [14,65]. Therefore, it is reasonable to assume that, as occurs in other tissues, the direct arrival of the nebulized platelet lysate molecules by the airway on lung cells will unleash a series of processes that affect the entire tissue, generating a biological environment favorable for healing. Relieving these processes of inflammatory reaction and tissue destruction also could lead to a modulated and less aggressive repair process, which, in addition to anti-fibrotic factors [58,66], could decrease or prevent pulmonary fibrosis. This prevention of fibrosis was already observed clinically in other tissues due to the reduction of inflammation and the repaired processes promoted by the PRP [67,68]. The cellular response observed in this work suggests that PRP nebulization does not affect the proliferation of lung fibroblasts, which would be involved in the repair processes.

The present work shows that PRP nebulization does not entail a detriment of its composition or its bioactivity on cell cultures, in terms of cellular proliferation. However, it can cause a minor water evaporation throughout the nebulization process, and this could explain the slightly higher growth factors and cytokine levels in the nebulized PL in comparison with the standard one. This difference was statistically significant in the case of PDGF, which was mainly due to the low standard deviation present between its replicates. In the proposed protocol, the PRP is not nebulized but the content is released after its activation or platelet lyase, which contains the effector biomolecules of plasma and platelets. In this way, subjecting the platelets to a process that could alter them is avoided, nebulizing only their content that does not seem to be degraded according to the data obtained. By nebulizing just the platelet lysate, the administration of proteins involved in the coagulation process is also avoided, preventing their possible action on the target tissues.

These results are preliminary, and further studies in additional relevant cell lines confirming the efficacy and safety of the nebulized platelet lysates could be useful. However, there are several preclinical and clinical studies that have demonstrated the therapeutic potential of administering PRP directly into the lungs (Table 3). In an in vitro study over airway epithelial cells subjected to pro-inflammatory stimuli, an increased viability of these cells was observed after the addition of HGF, which is one of the main active components of the PRP [69]. Mamoto et al. conducted in vivo studies evaluating the action of platelet lysate on lung tissue. In one of those studies, they demonstrated alveolar regeneration and stimulation of pulmonary vascularization after the application of platelet lysate in mice submitted to a pneumonectomy [70]. In other work of the same authors, they demonstrated that platelet lysate regulated the prevention of LPS-induced vascular leakage in the lungs of mice by suggesting a possible therapeutic approach for sepsis-induced ARDS as well as other diseases caused by abnormal vascular permeability [71]. Bronchial administration of PRP by instillation also proved to be a promising treatment in inflammatory pathologies. Dzyekanski et al. observed in an equine model of respiratory disease a significant improvement in clinical and cytological parameters seven days after its administration. These horses did not present cough, and their degree of tracheal mucus was significantly reduced as well as the relative neutrophil count. The authors concluded that PRP proved to be beneficial for lung inflammation and did not present biosecurity problems [72]. In addition to preclinical studies, a clinical study was conducted on burnt patients with airway disease [73]. This prospective study included 40 patients with 25–50% burns and inhalation lung injury. They were divided into two groups of 20 patients. The experimental group received aerosolized PRP particles in addition to the regular treatment. Biological improvements were observed in the upper airways of the PRP-treated patients compared to the control group. The mean extubation time was 7 ± 1 days in the PRP group compared to 14 ± 1 days in the control group, and it had a shorter hospital stay as well as a lower mortality rate. The authors concluded that the application of the nebulized PRP solution could be a beneficial therapeutic tool for the regeneration of the damaged pulmonary tree after the burn. Furthermore, the biosafety of the treatment was demonstrated. All these in vitro and in vivo studies reported no adverse effects. It is reasonable to expect that similar to any other drug, PRP could be toxic at high concentrations of its active ingredient, in this case platelets. However, cellular studies in which platelet concentrations were increased showed that above a certain level, the action of PRP decreased, but there was no evidence of toxicity [74,75]. These levels are far from what is used in clinical practice, and it has been shown in other medical fields with more experience with PRP that it is a safe product [14].

The platelet action is key in respiratory infections, and it can both improve the disease and aggravate the patient’s condition. Therefore, the balance in their functions is crucial in the evolution of the disease. Consequently, the direct administration of plasmatic and platelet biomolecules from healthy platelets into the lungs could preserve or recover such balance, helping alleviate the symptoms, and improving the evolution of the pathology and the appearance of sequelae.

## 4. Materials and Methods

### 4.1. Platelet-Rich Plasma Preparation

Blood was extracted from 4 healthy donors (3 female and 1 male) with ages ranging from 28 to 44 years old. Informed consent for sample donation was obtained from every donor from whom blood samples were extracted.

Platelet-rich plasma was obtained after blood centrifugation at 580× *g* for 8 min at room temperature into 3.8% (wt/v) sodium citrate containing 10 mL tubes. The plasma column located above the sedimented red blood cells, but not including the buffy coat, was collected in a tube. This plasma fraction preparation contained a concentration of platelets similar to the concentration of platelets compared with peripheral blood (depending on the platelet count and size as well as the hematocrit) and an absence of erythrocytes and leukocytes. The plasma obtained from each patient was activated adding CaCl_2_ (10% wt/vol), and once the clot was generated, it was squeezed and discarded to obtain the PRP lysate. Thus, the PRP used for this study was 11-00-11 (Table 1), following the recently defined classification system by Kon et al. [17]. Then, 2 mL of supernatant were separated and kept frozen for further use, and the rest of the lysate was subjected to nebulization.

### 4.2. Platelet-Rich Plasma Lysate Nebulization

**Figure 5:**
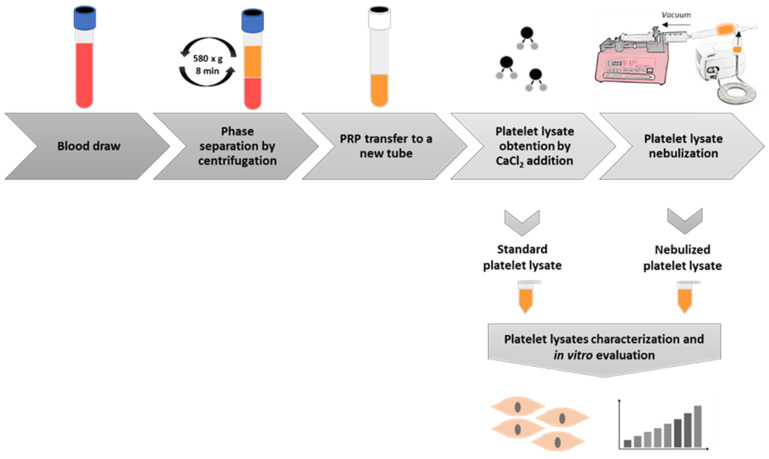
Schematic layout of the methodological protocol. Blood is drawn from healthy donors and it is centrifuged to obtain platelet-rich plasma (PRP), which is then activated by CaCl_2_ so that the growth factors present in the platelets are released. A fraction of this platelet lysate is transferred to a new tube, and the rest of the volume is nebulized and retrieved in a new tube. Then, both formulations, the standard and the nebulized ones, are assayed to characterize their growth factor and cytokine content and to evaluate their bioactivity potential on lung culture proliferation in vitro.

Platelet-rich plasma lysate was nebulized by an Apex mini plus compressor nebulizer. PRP was poured into the device reservoir, and a negative force of 1.5 mL/min was applied in a closed circuit by using a pump (Syringe Pump) in order to emulate aspiration. PRP was nebulized at a 0.2–2.3 mL/min rate, and aerosols containing particles of 0.5–5 microns were generated. These nebulized PRP was eventually condensed in the closed circuit and afterwards collected in a new tube. The whole methodological process is summarized in Figure 5.

### 4.3. Enzyme-Linked Immunosorbent Assay (ELISA)

In order to determine whether the nebulization process affects PRP content, the growth factor levels of standard and nebulized platelet lysates of four donors were measured by ELISA kits (Bio-techne; Minneapolis, MN, USA) in duplicate. The measured growth factors were CCL-11 (DTX00), VEGF (DVE00), TGF-β (DB100B), IGF-1 (DG100), HGF (DHG00B), and PDGF (DHD00C). All the protocols were conducted following manufacturer’s instructions. Growth factor levels were measured by absorbance, and concentrations were extrapolated by the corresponding calibration curve.

### 4.4. Cell Cultures and Culture Media

Normal human lung fibroblasts (CC-2512; Lonza, Basel, Switzerland) were employed for the in vitro evaluation of the nebulized PRP. They were kept in the incubator at 37 °C and 5% CO_2_. Cells were grown in fibroblast growth basal medium (CC-3131; Lonza, Basel, Switzerland) supplemented with insulin, human fibroblast growth factor, and gentamicin sulfate-amphotericin at 0.1% (*v*/*v*) each (CC-4126; Lonza, Basel, Switzerland), as recommended by the manufacturer. Additionally, either standard non-nebulized or nebulized PRP were added at a concentration of 10%. Serum-free medium was used as negative control.

### 4.5. Cell Viability Assay

In order to evaluate the biological activity of both standard and nebulized PLs, fibroblasts were incubated with either standard or nebulized platelet lysates of four donors, and a real-time follow-up of the cellular viability was registered along 96 h. Cellular viability was measured in triplicate in four independent experiments, by Realtime-Glo MT Cell Viability Assay (G9711; Promega, Fitchburg, WI, USA) that bases on the reducing potential of metabolically active cells that catalyze the conversion of a synthetic substrate into a luminescent product. The levels of luminescence that the detector reached can be considered proportional to the number of viable cells present in the assay. Then, the obtained growth curves with the different PRP formulations were compared to assess if the nebulization process affects PRP’s bioactivity.

### 4.6. Statistical Analysis

IBM SPSS^®^ Statistics v20 software (Armonk, NY, USA) was used for the statistical analysis. An unpaired *t*-test was used to compare the viability of cells cultured with different platelet lysate formulations and the different growth factor content of the two formulations.

## 5. Conclusions

The preliminary results indicated that the nebulization process of platelet lysate does not lead to a degradation of the analyzed molecules or a reduction of its bioactivity on cultured lung fibroblasts’ growth capacity. Considering platelets play a key role in COVID-19 and other respiratory infections, both initiating a defense response against the virus and, at the same time, contributing to a worsening of the disease, the administration of allogeneic healthy platelet lysates into the lungs could reestablish the biological condition of the lung to eliminate the infection and to avoid or reduce sequelae. In comparison with other therapies based on regenerative medicine, such as those using stem cells that are intravenously administered, the nebulized platelet lysate would directly target the damaged lung tissue, potentially achieving a quicker response in terms of anti-inflammatory action and tissue repair. The protocol presented in this work can be a first step for the research of this therapy both in animal models and in clinical trials. 

## Figures and Tables

**Table 1:** Summary of platelet-rich plasma characteristics.

**1. Platelet-Rich Plasma Preparation**	
Initial blood volume	10 mL
Anticoagulant	Sodium citrate 3.8% (wt/V)
System	Close
Centrifugation	Yes
* number*	* 1*
* speed*	* 580× g—8 min*
Final PRP volume	4 mL per subject
**2. Platelet-Rich Plasma Characteristics**	
PRP Type	11-00-11
Mean Platelet Volume (MPV)	9.6 ± 0.6 fL
Red Blood Cells	<0.01 × 10^6^/µL
White Blood Cells	<0.05 × 10^6^/µL
* Neutrophils*	---
* Lymphocytes*	---
* Monocytes*	---
* Eosinophils*	---
* Basophils*	---
Activation	CaCl_2_ (10% wt/vol)
**3. Application Characteristics**	
Dose	2%
Direct/Indirect	Direct
Cell line	Lung fibroblast
**4. Other Remarkable Platelet-Rich Plasma and Study Features**	
The product added to the cell cultures was the platelet lysate obtained after activation of PRP with calcium chloride (10%)	

**Table 2:** Potential effectors, targets, and actions of the active components of platelet lysates.

Effectors	Target	Action	References
HGF, IGF-1, PDGF, TGF-β	NF-kB cellular pathway	Anti-inflammatory effect	[14,59,65]
Platelet microparticles	Macrophages	Favoring the reparative phenotype M2	[55]
VEGF	Nrf2 cellular pathway	Preventing oxidative damage	[56]
Sphingosine-1	endothelial cells	Preserving alveolocapillary barrier	[43]
PDGF, TGF-β, EGF, FGF-2, CTGF and other growth factors	Lung cell populations: fibroblast, endothelial and epithelial cells, alveolar Type II Cells	Cell migration and proliferation, and tissue repair	[57,62]
VEGF-A/TGF-β1	Fibroblast	Antifibrotic effect	[58,66]

**Table 3:** Preclinical and clinical studies on platelet-rich plasma administration in lung tissue.

Type of Study	Target Cell/Tissue	Route of Administration	Effect	Reference
*In vitro* study	Airway epithelial cells	HGF at different concentrations in cell culture	Anti-apoptotic effect against pro-inflammatory stimuli	[69]
*In vivo* study	Lung tissue in mice subjected to pneumonectomy	Platelet lysate administered intraperitoneally	Stimulation of adult mouse lung vascular and alveolar regeneration	[70]
*In vivo* study	Lung tissue in mice treated with LPS to generate edema and ARDS	Platelet lysate administered intraperitoneally	Prevention of LPS-induced vascular leakage in lungs	[71]
*In vivo* study	Horses with inflammatory airway disease	Intrabronchial instillation	Significative improvement of horses, with a reduction of the mucus grade and neutrophil amount	[72]
Clinical study	Patients with inhalation lung injury	Aerosolized PRP	Lower extubation time, hospital stay and mortality rate	[73]

## Data Availability

The data presented in this study are available within the article.

## Abbreviations

SARS-CoV-2	Severe Acute Respiratory Syndrome Coronavirus 2
COVID-19	Coronavirus Disease 2019
ARDS	Acute Respiratory Distress Syndrome
PRP	Platelet-Rich Plasma
PL	Platelet Lysate
CCL-11	C-C Motif Chemokine Ligand 11 (Eotaxin)
CTGF	Connective Tissue Growth Factor
HGF	Hepatocyte Growth Factor
IGF-1	Insulin-Like Growth Factor 1
PDGF	Platelet-Derived Growth Factor
TGF-β	Transforming Growth Factor Beta
VEGF	Vascular Endothelial Growth Factor
GPiB	Glycoprotein Ib
GPVI	Glycoprotein VI
PRR	Pattern Recognition Receptors
PAMP	Pathogen-Associated Molecular Pattern
DAMP	Damage-Associated Molecular Pattern
TLR	Tool-Like Receptor
CLR	C-type Lectin Receptor
DC-SIGN	Dendritic Cell-Specific ICAM3-Grabbing Non-Integrin
ACE-2	Angiotensin Converting Enzyme 2
NET	Neutrophil Extracellular Trap
TMPRSS2	Transmembrane protease, serine 2
ITGA2B	Integrin alpha-IIB
CXCL4	Platelet Factor 4
IL-1B	Interleukin 1-β
MSC	Mesenchymal Stem Cells
GM-CSF	Granulocyte-Macrophage Colony-Stimulating Factor
ELISA	Enzyme-Linked Immunoabsorbent Assay
MCP-1	Monocyte Chemoattractant Protein-1
RNA	Ribonucleic Acid
MPV	Mean Platelet Volume
